# Expression of MAF bZIP transcription factor B protects against ulcerative colitis through the inhibition of the NF‐κB pathway

**DOI:** 10.1002/iid3.1372

**Published:** 2024-08-22

**Authors:** Jingwen Li, Qingmin Li, Wei Ma, Yongsheng Zhang, Xiaonan Li

**Affiliations:** ^1^ Department of Gastroenterology Shandong Provincial Hospital Affiliated to Shandong First Medical University Jinan Shandong China; ^2^ Department of General Practice Shandong Provincial Hospital Affiliated to Shandong First Medical University Jinan Shandong China; ^3^ Department of Medicine Zhangqiu District Gaoguanzhai Community Health Service Center Jinan Shandong China

**Keywords:** colitis, epithelial barrier impairment, inflammation, MAFB, NF‐κB pathway

## Abstract

**Purpose:**

The aim of this study was to explore whether MAF bZIP transcription factor B (MAFB) might alleviate ulcerative colitis (UC) in dextran sulfate sodium (DSS)‐induced mice and LPS‐induced IEC‐6 cells.

**Methods:**

UC in vivo and in vitro model was established by using DSS and LPS, respectively. The mice body weight and disease activity index (DAI) score were recorded daily, and colon length was measured. Moreover, the permeability was evaluated utilizing a fluorescein isothiocyanate dextran (FITC‐Dextran) probe. Histopathological changes of DSS‐induced colitis mice was assessed utilizing H&E staining. Next, qRT‐PCR was performed to detect IL‐1β, IL‐6, TNF‐α, and IL‐10 level in in vivo and in vitro. Furthermore, the level of MDA, SOD, CAT, and GSH were evaluated in colon tissues. Besides, the expressions of tight junction proteins and NF‐κB pathway relative proteins were examined in colitis mice and IEC‐6 cells using western blot, immunohistochemistry and immunofluorescence.

**Results:**

MAFB level was downregulated in DSS‐induced colitis mice. Moreover, the upregulation of MAFB protected mice from DSS‐induced colitis by suppressing DSS‐induced inflammation, oxidative stress, and intestinal barrier impairment. We also demonstrated that the upregulation of MAFB inactivated NF‐κB pathway in DSS‐caused colitis mice. Subsequently, we observed that MAFB upregulation could inhibit LPS‐caused epithelial barrier impairment and inflammation in IEC‐6 cells. Additionally, MAFB overexpression could suppress the activation of NF‐κB pathway in IEC‐6 cells.

**Conclusion:**

The upregulation of MAFB could protect against UC via the suppression of inflammation and the intestinal barrier impairment through inhibiting the NF‐κB pathway.

## Highlights

- MAFB inhibits inflammation and intestinal barrier impairment in colitis mice.
- MAFB inactivates NF‐κB signaling in colitis mice.
- MAFB inhibits epithelial barrier injury and inflammation in LPS‐induced IEC‐6 cells.
- MAFB inactivates NF‐κB signaling in LPS‐treated IEC‐6 cells.

## Graphical abstract

The upregulation of MAFB protects against ulcerative colitis via the suppression of inflammation, oxidative stress and the intestinal barrier impairment through inhibiting the NF‐κB pathway.

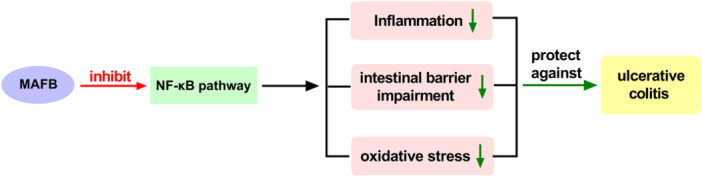

## 1 INTRODUCTION

Ulcerative colitis (UC), the most common subtype of inflammatory bowel disease (IBD), is a chronic, relapsing and remitting disorder in the gastrointestinal tract.^1^, ^2^, ^3^ Because chronic intestinal inflammation has a pro‐tumor effect, the risk of colorectal cancer in patients with colitis is significantly increased if left untreated.^4^ The prevalence of IBD is estimated at 7 million all over the world.^5^ At present, UC is generally treated with anti‐inflammatory drugs and immunosuppressive agents, but long‐term uses of these drugs and agents have shown various adverse effects.^6^, ^7^, ^8^ Hence, finding a novel safe and effective therapeutic method for UC is necessary.

MAF bZIP transcription factor B (MAFB) is a kind of the large musculoaponeurotic fibrosarcoma oncogene (Maf) transcription factor family which can encode a leucine‐zipper transcription factor.^9^ Recent evidence has indicated that the elevated MAFB expression is found in anti‐inflammatory‐type macrophages.^10^ Singh et al.^11^ manifests MAFB overexpression in immune system and islets is critical for inhibiting islet inflammation. Sun et al.^12^ have proved that MAFB could protect against allergic rhinitis through the suppression of inflammation via modulating epithelial barrier function. Besides, MAFB overexpression in podocytes is proved to prevent the progression of diabetic nephropathy.^13^ However, the function of MAFB in colitis has not been evaluated till date.

In this study, we aimed to explore the effect of MAFB on UC and its potential mechanisms. We observed that MAFB was lowly expressed in dextran sulfate sodium (DSS)‐caused colitis mice. Additionally, MAFB upregulation could protect against UC via the suppression of inflammation and the intestinal barrier impairment through inhibiting the NF‐κB pathway. Taken together, this research manifests an important role of MAFB in UC and represents a new therapeutic target against UC.

## 2 MATERIALS AND METHODS

### 2.1 Animals

Male C57BL/6 mice (8‐week‐old; body weight, 20–25 g) were supplied by Pengyue Experimental animal Breeding and housed in a temperature‐controlled room (22 ± 2°C) on a 12‐h circadian rhythm, and given a regular chow and water ad libitum.

### 2.2 Animal model and treatment

Mice were randomly allocated into separate experimental groups of five mice in each: Control, DSS, DSS + Vector and DSS + MAFB group. Animals were given 3% (w/v) DSS (MP Biomedicals) in daily drinking water for 7 days to cause the colitis. Two days before DSS treatment, the mice in DSS + Vector group or DSS + MAFB group were respectively injected with NC lentivirus or MAFB‐overexpression lentivirus (5 × 10^8^ TU/mL) via the tail vein. Mice body weight, stool consistency as well as rectal bleeding were recorded once a day for assess disease activity index (DAI) as reported previously.^14^ The mice were killed by cervical dislocation under anesthesia, and then the colon length was recorded and stored at −80°C.

### 2.3 In vivo permeability assay

At Day 6 after DSS administration, the mice were given with FITC‐dextran (0.6 mg/g body weight, Sigma‐Aldrich) orally. After 4 h, blood was collected and subsequently centrifuged at 4°C, 3000 rpm for 10 min. Finally, FITC concentration in serum was measured utilizing a multifunctional microplate reader.

### 2.4 Hematoxylin and eosin (H&E) staining and immunohistochemistry staining

The colon segments were fixed in 4% paraformaldehyde (PH 7.4) overnight and dehydrated in a graded ethanol series, After embedding in paraffin, the consecutive sections with 5‐μm thickness were prepared and then stained utilizing H&E. At last, the histopathological changes were evaluated via employing a light microscope. Besides, histological score was assessed based on the criteria reported previously.^15^

For immunohistochemistry, the paraffin‐embedded colon tissues were soaked in 3% hydrogen peroxide for 30 min to inactivate the endogenous peroxidase. Next, the sections were incubated with anti‐MAFB antibody (#K009896P, 1:50; Solarbio) at 4°C overnight and counterstained utilizing DAB chromogenic reagent and hematoxylin. In the end, the samples were photographed with a light microscope.

### 2.5 Quantitative real‐time PCR (qRT‐PCR)

Total RNA from colon tissues and cells was isolated employing TRIzol (Sigma‐Aldrich) based on the provider protocol. RNA was reversed transcribed into cDNA utilizing the ImProm‐II Reverse Transcription System (Promega). Quantitative PCR was performed applying the FastStart SYBR Green reagent (Sigma‐Aldrich) with primers for MAFB‐mouse (F ATGGCCGCGGAGCTGAGCATG; R CATGCTCAGCTCCGCGGCCAT), IL‐1β‐mouse (F ATGCCACCTTTTGACAGTGAT; R AAGGTCCACGGGAAAGACAC), IL‐1β‐rat (F TGTCTGACCCATGTGAGCTG; R TTTGGGATCCACACTCTCCA), IL‐6‐mouse (F CCAGTTGCCTTCTTGGGACT; R GAATTGCCATTGCACAACTCT), IL‐6‐rat (F TCCGGAGAGGAGACTTCACA; R TTGCCATTGCACAACTCTTTTC), TNF‐α‐mouse (F CCCACGTCGTAGCAAACCA; R ACAAGGTACAACCCATCGGC), TNF‐α‐rat (F GTAGCCCACGTCGTAGCAAA; R AAATGGCAAATCGGCTGACG), IL‐10‐mouse (F GGTTGCCAAGCCTTATCGGA; R GACACCTTGGTCTTGGAGCTTA), IL‐10‐rat (F TTCCCTGGGAGAGAAGCTGA; R GACACCTTTGTCTTGGAGCTTA), β‐actin‐mouse (F CCACTGTCGAGTCGCGTCC; R GTCATCCATGGCGAACTGGTG), and β‐actin‐rat (F GCCTTCCTTCCTGGGTATGG; R AATGCCTGGGTACATGGTGG).

### 2.6 Protein isolation and western blot

The proteins from colon tissues and cells were isolated by using the RIPA buffer (Beyotime). Next, the protein concentration was estimated via employing a BCA kit (Beyotime). Afterwards, the denatured proteins were resolved on 10% SDS‐PAGE and transferred onto PVDF membranes (Millipore). The 5% nonfat skim milk was employed to block the membranes in the Tris‐buffered saline. After that, the membrane was incubated in the corresponding primary antibodies in 3% bovine serum albumin (MAFB, 1:500, #K009896P; Solarbio; Occludin, 1:500, #91131; Cell Signaling; ZO‐1, 1:1000, #21773‐1‐AP; Proteintech; p‐IKK, 1:300, #36214; Cell Signaling; IKK, 1:1000, #ab32041; Abcam; p‐IκBα, 1:500, #ab133462; Abcam; IκBα, 1:1000, #ab32518; Abcam; p‐NF‐κB, 1:500, #3033; Cell Signaling; NF‐κB, 1:1000, #8242; Cell Signaling; GAPDH, 1:2000, #ab9485; Abcam; β‐actin, 1:3000, #ab8227; Abcam) overnight at 4°C with shaking. Next, bands were revealed by an ECL kit (Beyotime) followed by the incubation of secondary antibody for 60 min.

### 2.7 Measurement of malondialdehyde (MDA), reduced glutathione (GSH), catalase (CAT), and superoxide dismutase (SOD) level

Based on the provider protocol, the level of MDA, GSH, CAT, and SOD in colon tissues was estimated via utilizing a MDA content assay kit (Solarbio), a GSH content assay kit (Solarbio), a CAT activity assay kit, and a SOD activity assay kit (Solarbio), respectively.

### 2.8 Cell culture and treatment

Rat intestinal epithelial cell line IEC‐6 cells were purchased from the ATCC and maintained in DMEM medium (Gibco) containing the mixture of 10% FBS (HyClone) and 1% antibiotics (Solarbio) at 37°C with a humidified atmosphere of 5% CO_2_. IEC‐6 cells were treated using LPS (50 μg/mL, Solarbio) for 12 h to simulate the injury of intestinal epithelial cells. Besides, before LPS treatment, pcDNA‐NC or pcDNA‐MAFB was transfected into IEC‐6 cells for 24 h utilizing Lipofectamine 3000 (Invitrogen) based on the provider protocol.

### 2.9 Immunofluorescence

IEC‐6 cells were permeabilized by 0.3% Triton X‐100 and fixed with 4% fresh paraformaldehyde at room temperature followed by cultivation with anti‐Occludin (#91131, 1:100; Cell Signaling) and anti‐ZO‐1 (#21773‐1‐AP, 1:100; Proteintech) at 4°C overnight. Next, the slides were subjected to 60‐min incubation in Alexa Fluor 488‐conjugated goat anti‐rabbit IgG secondary antibody (Cell Signaling) under the room temperature. Subsequently, DAPI (Beyotime) was employed for dying nucleus for 20 min. In the end, the coverslips were observed under a Eclipse Ni‐U fluorescence microscope.

### 2.10 Statistical analysis

Data analyses were performed employing GraphPad Prism 8.0 and SPSS 22.0 Differences were analyzed using unpaired Student's *t* test or one way ANOVA with Tukey's post hoc test. Results are shown as mean ± SD. A value of *p* < .05 was considered significant.

## 3 RESULTS

### 3.1 MAFB level was downregulated in DSS‐induced colitis of mice

**Figure 1:**
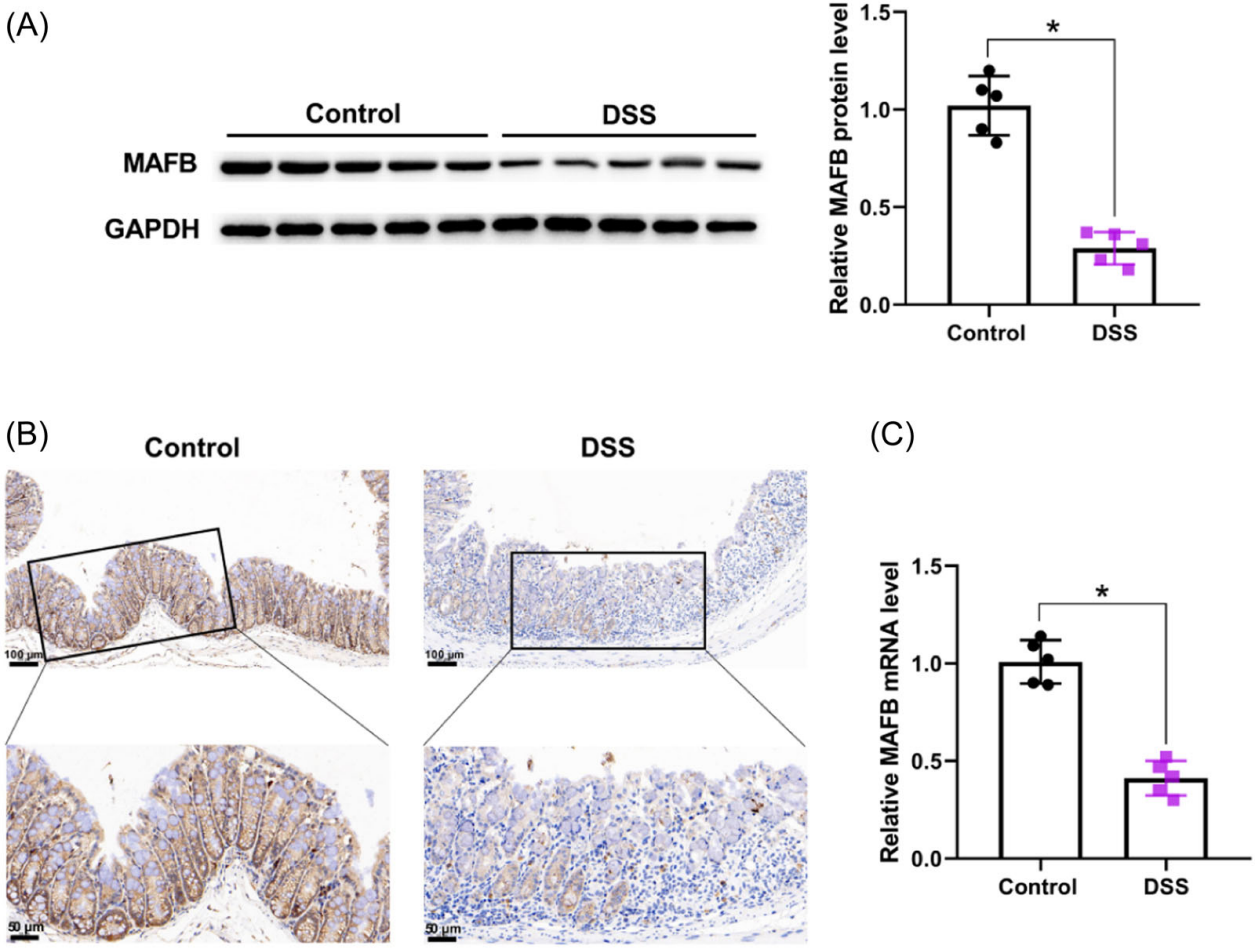
MAFB level was downregulated in DSS‐induced colitis mice. Western blot (A), immunohistochemistry staining (B), and qRT‐PCR (C) for measuring MAFB expression in DSS group and control group mice. Scale bar: 100 μm (×100), 50 μm (×200). Data were displayed as mean ± SD. *N* = 5 per group. Statistical analyses were performed using unpaired Student's *t* test. *p* < .05 was considered to indicate a statistically significant difference. *p < .05 versus Control group.

To define the relevance between MAFB and UC, we examined MAFB level in DSS‐induced colitis in C57BL/6J mice. The results of western blot manifested that MAFB expression was lower in DSS group mice than that in Control group mice (Figure 1A). Moreover, immunohistochemistry staining results demonstrated that MAFB protein expression was significantly downregulated in colonic epithelia of DSS‐induced colitis mice (Figure 1B). In addition, qRT‐PCR also demonstrated that the mRNA level of MAFB was markedly reduced in DSS‐induced colitis of mice (Figure 1C). Collectively, these results indicated that MAFB level was downregulated in DSS‐induced colitis of mice.

### 3.2 The upregulation of MAFB protected mice from DSS‐caused colitis

**Figure 2:**
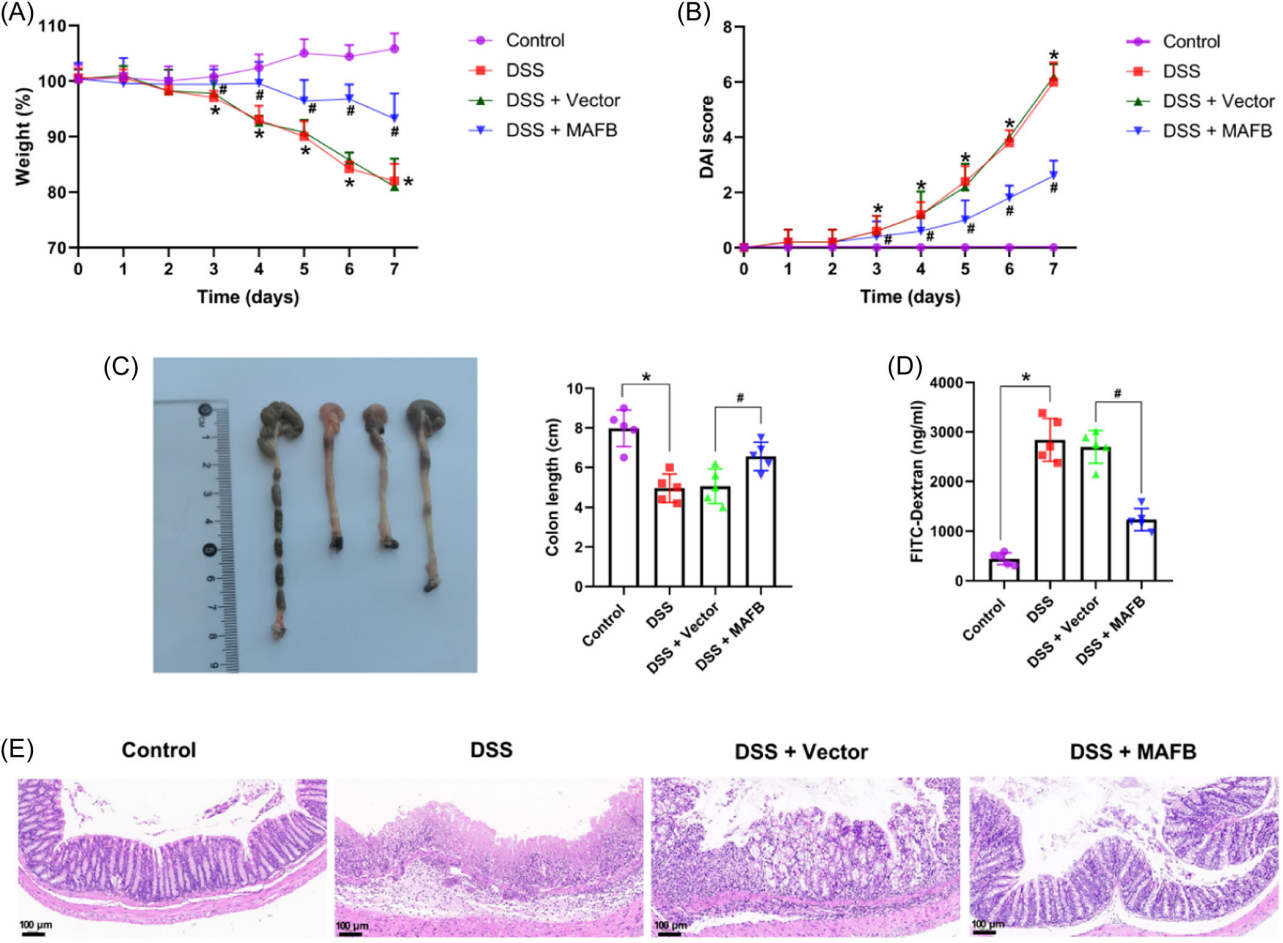
The upregulation of MAFB protected mice from DSS‐induced colitis. Following the treatment of DSS and MAFB‐overexpression lentivirus, mice body weight (A) and DAI (B) were observed once a day; (C) colon length; (D) fluorescein isothiocyanate (FITC)‐dextran measurement in serum from DSS‐induced mice; (E) histopathological changes of colon tissues was assessed utilizing H&E staining. Scale bar: 100 μm (×100). Data were displayed as mean ± SD. (A and B) *N* = 10 per group; (C–E) *N* = 5 per group. Statistical analyses were performed using one way ANOVA. *p* < .05 was considered to indicate a statistically significant difference. *p < .05 versus Control group; ^#^ *p* < .05 versus DSS + Vector group.

The decreased weight loss and the increased DAI were observed in DSS group compared with control group, but significantly reversed by MAFB upregulation (Figure 2A,B). As seen in Figure 2C, the colon length was decreased in DSS group mice compared with control group mice. When compared with DSS + Vector group, the colon length was markedly increased in DSS + MAFB group (Figure 2C). Moreover, we found that DSS group mice had significantly higher FITC level, whereas MAFB overexpression revealed an improvement in gut permeability (Figure 2D). H&E staining manifested that the severe enteric mucosal injury was found in colitis mice (Figure 2E). However, MAFB upregulation significantly improved the severe enteric mucosal injury (Figure 2E). Together, these data suggested that the upregulation of MAFB could protect mice from DSS‐caused colitis.

### 3.3 The upregulation of MAFB suppressed DSS‐induced inflammation, oxidative stress, and intestinal barrier impairment in colitis mice

**Figure 3:**
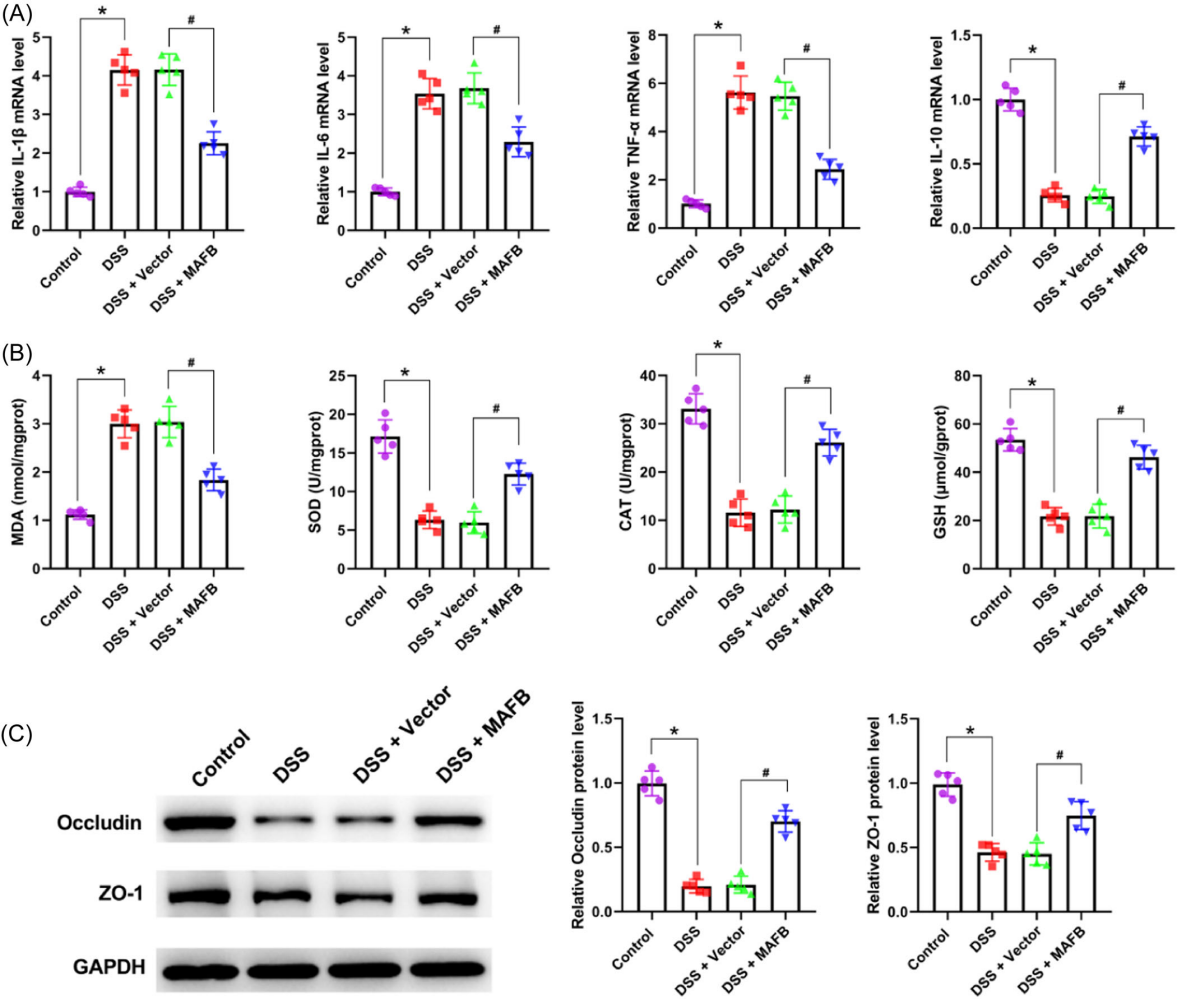
The upregulation of MAFB suppressed DSS‐induced inflammation, oxidative stress, and intestinal barrier impairment in colitis mice. Following the treatment of DSS and MAFB‐overexpression lentivirus, (A) the IL‐1β, IL‐6, TNF‐α, and IL‐10 level in colon tissues was examined by employing qRT‐PCR; (B) the levels of MDA, SOD, CAT, and GSH were evaluated in colon tissues; (C) western blot was employed to examine Occludin and ZO‐1 expression in colon tissues. Data were displayed as mean ± SD. *N* = 5 per group. Statistical analyses were performed using one way ANOVA. *p* < .05 was considered to indicate a statistically significant difference. *p < .05 versus Control group; ^#^ *p* < .05 versus DSS + Vector group. DSS, dextran sulfate sodium.

As shown in Figure 3A, the expressions of IL‐1β, IL‐6, and TNF‐α were significantly increased in DSS‐treated mice relative to control group mice, while IL‐10 expression was decreased. Besides, the above phenomenon were reversed by the upregulation of MAFB (Figure 3A). Moreover, we also observed that the increased MDA level, and the decreased SOD, CAT, and GSH levels caused by DSS were neutralized by MAFB upregulation (Figure 3B). Overall, these results demonstrated that the upregulation of MAFB could suppress DSS‐induced inflammation and oxidative stress in colitis mice. As seen in Figure 3C, the reduced levels of Occludin and ZO‐1 caused by DSS were markedly reversed after the treatment of MAFB overexpression, proving that the upregulation of MAFB could inhibit DSS‐induced intestinal barrier impairment in colitis mice.

### 3.4 The upregulation of MAFB inhibited the activation of NF‐κB pathway in DSS‐induced colitis mice

**Figure 4:**
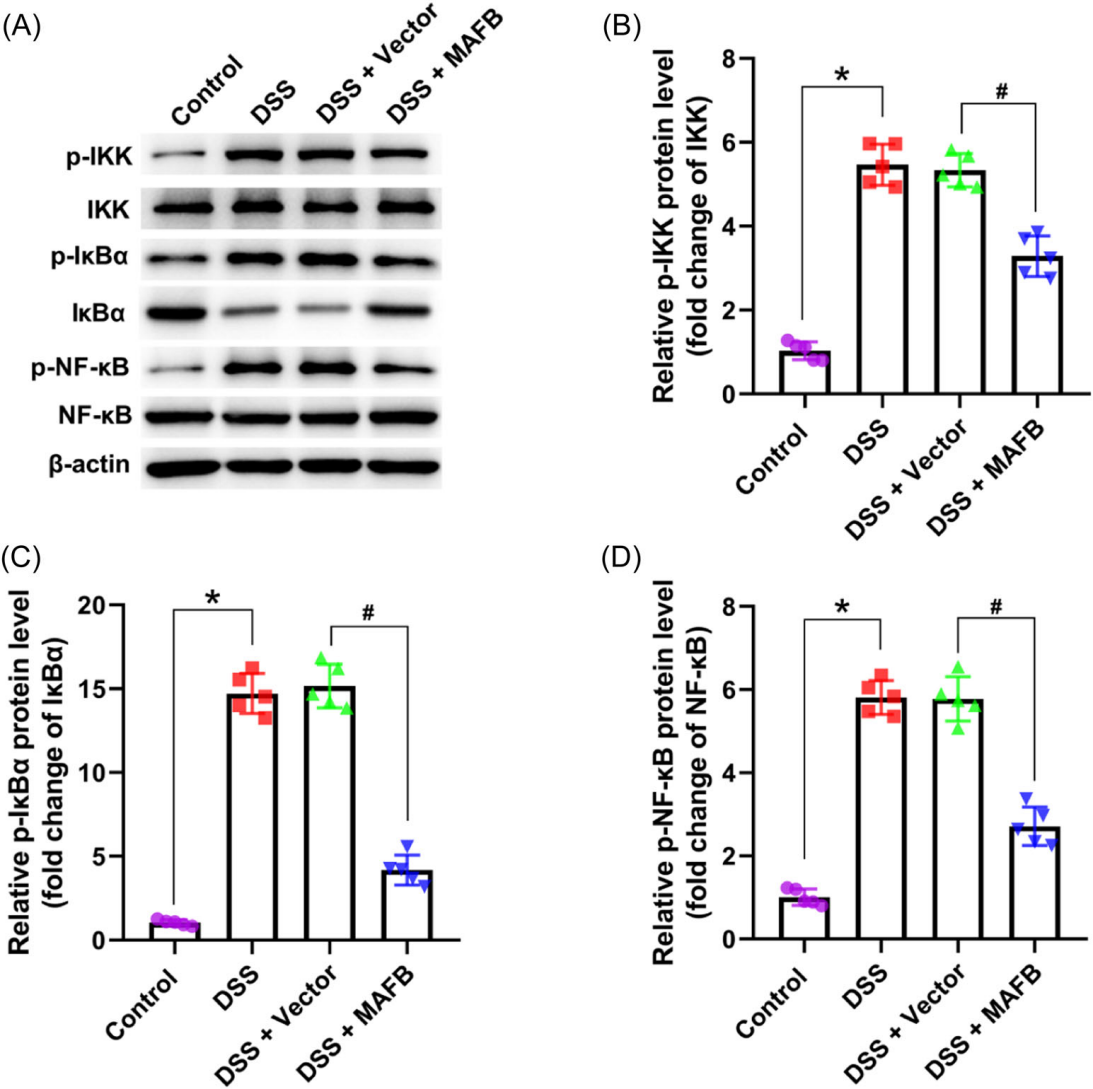
The upregulation of MAFB inactivated NF‐κB signaling in DSS‐induced colitis mice. Following the treatment of DSS and MAFB‐overexpression lentivirus, (A) the representative western blot bands was showed; (B) the quantitative data for p‐IKK/IKK was determined in colon tissues; (C) the quantitative data for p‐IκBα/IκBα was determined in colon tissues; (D) the quantitative data for p‐NF‐κB/NF‐κB was determined in colon tissues. Data were displayed as mean ± SD. *N* = 5 per group. Statistical analyses were performed using one way ANOVA. *p* < .05 was considered to indicate a statistically significant difference. *p < .05 versus Control group; ^#^ *p* < .05 versus DSS + Vector group. DSS, dextran sulfate sodium.

The expressions of p‐IKK/IKK, p‐IκBα/IκBα, and p‐NF‐κB/NF‐κB were notably elevated in DSS group mice compared with control group mice (Figure 4A–D). On the contrary, when compared with DSS + Vector group, the expression of p‐IKK/IKK, p‐IκBα/IκBα, and p‐NF‐κB/NF‐κB significantly decreased in DSS + MAFB group (Figure 4A–D). These results demonstrated that the upregulation of MAFB could inactivate NF‐κB signaling in DSS‐caused colitis mice.

### 3.5 The upregulation of MAFB suppressed LPS‐induced epithelial barrier impairment in IEC‐6 cells

**Figure 5:**
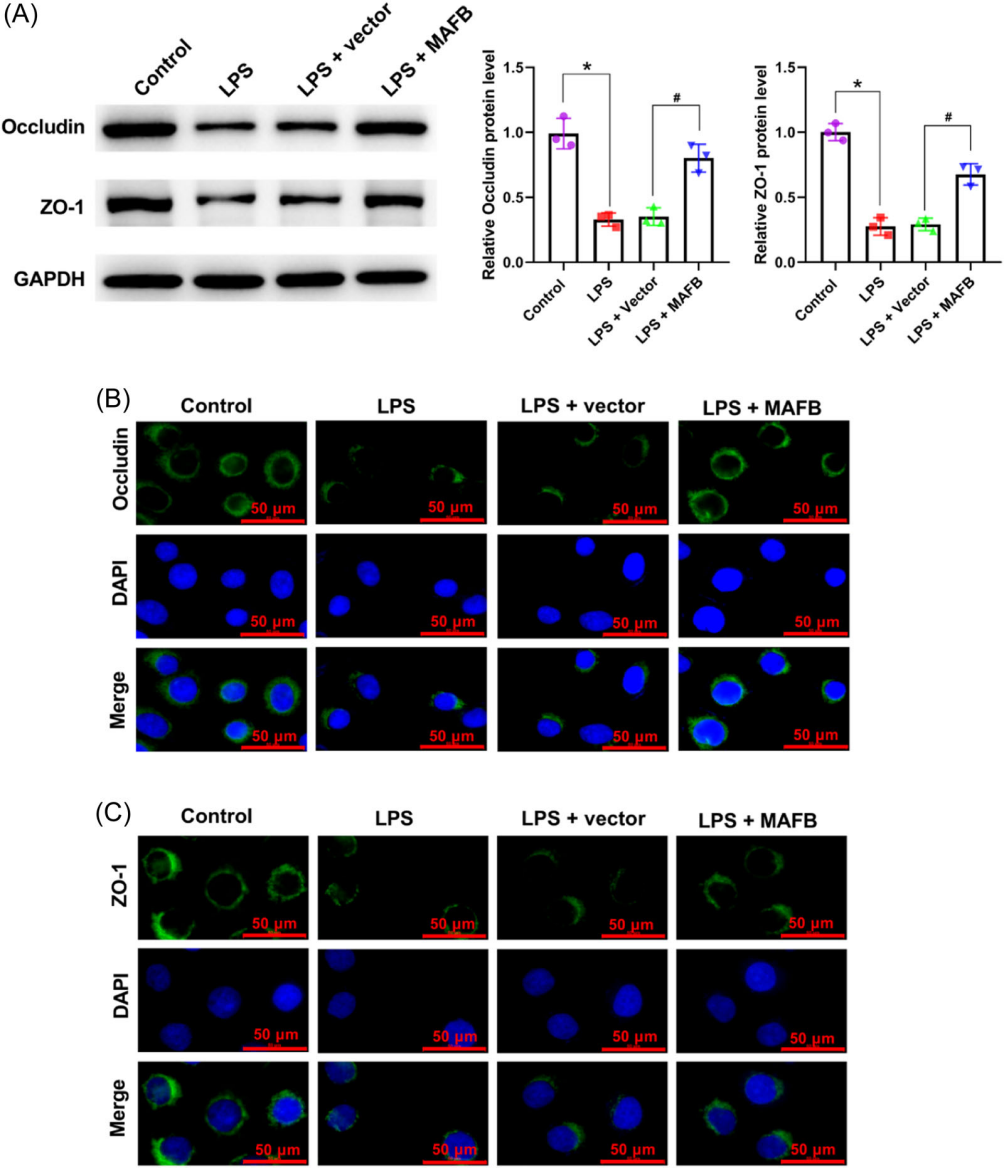
The upregulation of MAFB suppressed LPS‐caused epithelial barrier impairment in IEC‐6 cells. Following the treatment of LPS and MAFB‐overexpression vector (A), western blot was employed to examine Occludin and ZO‐1 expression in IEC‐6 cells; immunofluorescence was employed to determine Occludin (B), and ZO‐1 expression (C) in IEC‐6 cells. Scale bar: 50 μm (×400). Data were displayed as mean ± SD. *N* = 3 per group. Statistical analyses were performed using one way ANOVA. *p* < .05 was considered to indicate a statistically significant difference. *p < .05 versus Control group; ^#^ *p* < .05 versus LPS + Vector group.

The reduced expression of Occludin and ZO‐1 induced by LPS in IEC‐6 cells was significantly reversed by MAFB overexpression (Figure 5A). Besides, the above effect of MAFB overexpression on Occludin and ZO‐1 expression was further verified by immunofluorescence (Figure 5B,C). Overall, these data testified that the upregulation of MAFB suppressed epithelial barrier impairment in LPS‐treated IEC‐6 cells.

### 3.6 The upregulation of MAFB suppressed LPS‐induced inflammation and inactivated NF‐κB signaling in IEC‐6 cells

**Figure 6:**
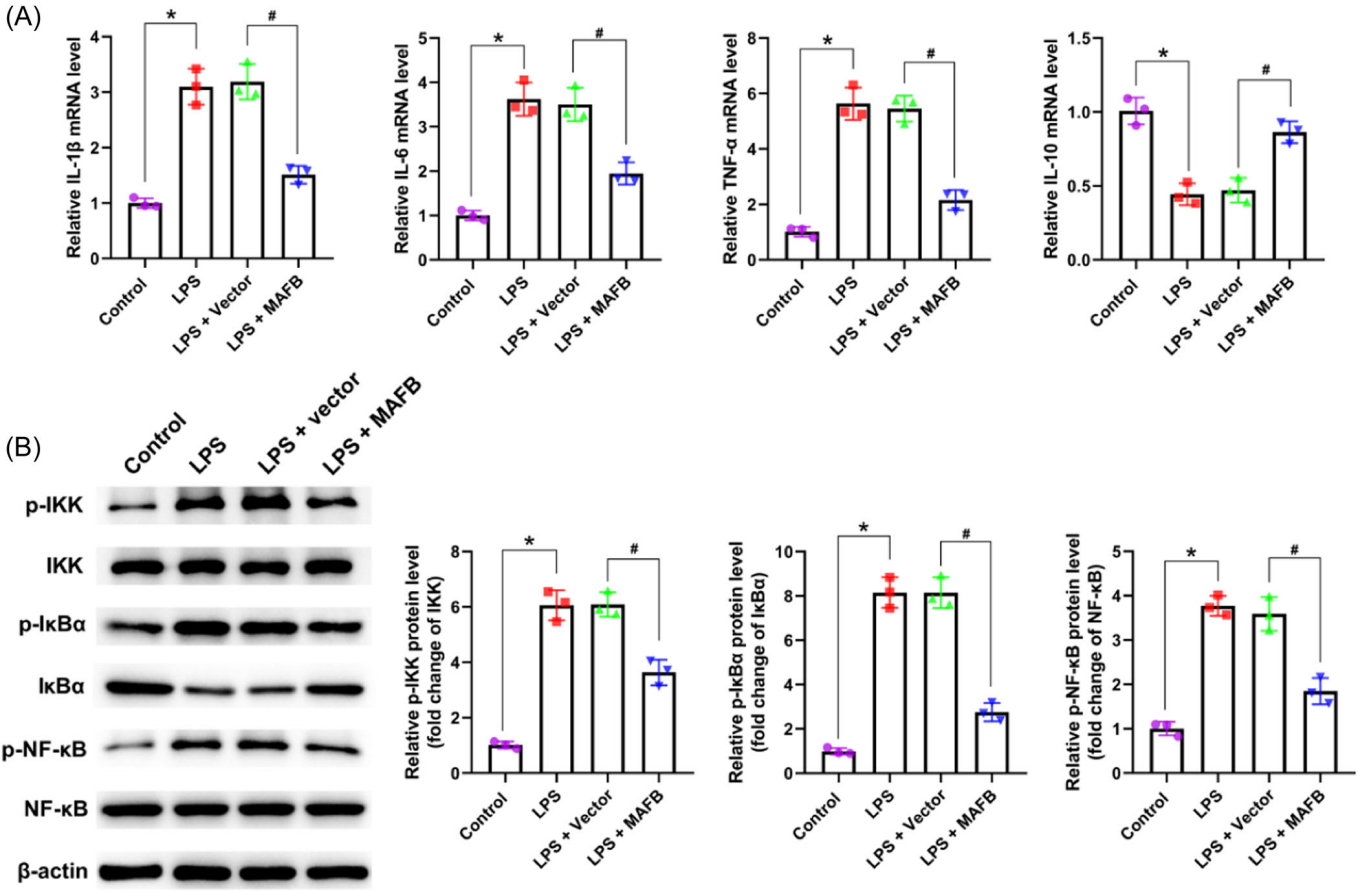
The upregulation of MAFB suppressed LPS‐induced inflammation and the activation of NF‐κB pathway in IEC‐6 cells. Following the treatment of LPS and MAFB‐overexpression vector, (A) the IL‐1β, IL‐6, TNF‐α, and IL‐10 level in IEC‐6 cells was evaluated employing qRT‐PCR; (B) p‐IKK, IKK, p‐IκBα, IκBα, p‐NF‐κB, and NF‐κB level in IEC‐6 cells was examined utilizing western blot. Data were displayed as mean ± SD. *N* = 3 per group. Statistical analyses were performed using one way ANOVA. *p* < .05 was considered to indicate a statistically significant difference. *p < .05 versus Control group; ^#^ *p* < .05 versus LPS + Vector group.

The results of Figure 6A manifested that the increased IL‐1β, IL‐6, and TNF‐α level, and the decreased IL‐10 level induced by LPS in IEC‐6 cells were reversed by the upregulation of MAFB. Moreover, the expressions of p‐IKK/IKK, p‐IκBα/IκBα, and p‐NF‐κB/NF‐κB were dramatically increased in LPS‐induced IEC‐6 cells (Figure 6B). Importantly, we observed that MAFB overexpression significantly reversed the elevated p‐IKK/IKK, p‐IκBα/IκBα, and p‐NF‐κB/NF‐κB levels in IEC‐6 cells (Figure 6B). These findings revealed that the upregulation of MAFB could suppress LPS‐induced inflammation and the activation of NF‐κB pathway in IEC‐6 cells.

## 4 DISCUSSION

In recent years, the mortality rate of UC in Asian region has been increasing year by year.^16^ A deeper understanding of UC pathogenesis is urgently needed to optimize its treatment and decrease the burden of disease. In our study, we proved that the upregulation of MAFB could protect against UC via the suppression of inflammation and the intestinal barrier impairment through inhibiting NF‐κB signaling.

MAFB is considered to be an important transcriptional activator of anti‐inflammatory cytokine genes.^11^ Zhang et al.^17^ have revealed that MAFB expression was notably decreased in SH‐SY5Y cells following oxygen glucose deprivation/reoxygenation, and also demonstrated that MAFB could suppress inflammation response in cerebral ischemia‐reperfusion injury. Our study observed that MAFB expression was downregulated in DSS‐caused colitis mice. Besides, the loss of MAFB has been reported to impair anti‐inflammatory polarization of macrophages.^18^ Inflammatory response and oxidative stress frequently happen during the progression of UC, which can explain the occurrence and aggravation of UC caused by inflammatory infiltration and oxidative injury.^19^, ^20^ With the onset of UC, the secretion of TNF‐α, IL‐1β as well as other inflammatory cytokines is elevated in the inflammatory intestinal mucosa, that is associated with UC severity.^21^, ^22^ Oxidative stress is often accompanied by inflammatory response and is also considered to be one of the important factors in the occurrence and development of IBD.^23^ IBD can cause an overproduction of reactive oxygen species (ROS) and free radicals with accompanying insufficiency of endogenous antioxidant enzyme systems resulting in elevated oxidative stress.^24^ Usually, antioxidant enzyme systems includes SOD, CAT, and GSH.^25^ Previous study confirms that MAFB could inhibit mitochondrial ROS production.^26^ Morito et al.^13^ have reported that MAFB overexpression in podocytes could ameliorate diabetic nephropathy through regulating antioxidative stress enzyme. Our findings also verified that MAFB upregulation could suppress inflammation and oxidative stress in DSS‐treated colitis mice and in LPS‐treated IEC‐6 cells. Epithelial barrier damage is discovered to induce disproportionate immune responses, which can further lead to the chronic inflammation.^27^ Increasing studies have suggested that the intestinal epithelial damage is the typical feature of UC.^14^, ^28^ In addition, the impairment of epithelial barrier is often related to tight junction proteins expressions, including ZO‐1 and Occludin.^29^, ^30^ Our findings demonstrated that the upregulation of MAFB could improve epithelial barrier impairment in UC mice and cell model through upregulating ZO‐1 and Occludin expression.

NF‐κB is considered to be an important regulator of inflammation, innate immunity, infections, and tissue integrity.^31^, ^32^ Various stimuli such as pro‐inflammatory enzymes and cytokines modulates the activation of TAK1‐mediated IκB kinase (IKK) complex.^33^ Subsequently, the activated IKK will further phosphorylate IκBα and causes the ubiquitin‐dependent IκBα degradation.^34^ More and more research have associated with the dysregulation of NF‐κB pathway to various critical medical conditions.^35^, ^36^, ^37^, ^38^ Interestingly, with emerging evidence, NF‐κB pathway is also confirmed to play an important role in UC.^39^, ^40^, ^41^, ^42^ Besides, NF‐kB activating in the intestinal mucosa causes the expressions of pro‐inflammatory as well as adhesion molecules.^43^ Importantly, Tian et al.^44^ have proved that MAFB/Msr1/PI3K‐Akt/NF‐κB signaling can participate in the development of subarachnoid hemorrhage. In this study, we manifested that the upregulation of MAFB could inactivate NF‐κB signaling in UC mice and cell model.

In summary, we first observed that MAFB expression was downregulated in DSS‐induced colitis mice. In addition, we verified that the upregulation of MAFB could protect against UC via the suppression of inflammation and the intestinal barrier impairment through inhibiting the NF‐κB pathway. Our work lays a theoretical foundation for further study of targeted therapy of UC.

## AUTHOR CONTRIBUTIONS

*Conception and design*: Jingwen Li and Xiaonan Li. *Administrative support*: Xiaonan Li. *Provision of study materials*: Qingmin Li, Wei Ma, and Yongsheng Zhang. *Collection and assembly of data*: Jingwen Li, Wei Ma, and Yongsheng Zhang. *Data analysis and interpretation*: Qingmin Li and Xiaonan Li. *Manuscript writing*: Jingwen Li, Qingmin Li, and Xiaonan Li. *Final approval of the manuscript*: Jingwen Li, Qingmin Li, Wei Ma, Yongsheng Zhang, and Xiaonan Li.

## CONFLICT OF INTEREST STATEMENT

The authors declare no conflict of interest.

## ETHICS STATEMENT

This study was approved by the Animal Ethics Committee of Shandong Provincial Hospital Affiliated to Shandong First Medical University (No. 2022‐0164).

## Data Availability

The data that supported the findings of this study are available from the corresponding author upon reasonable request.
